# Cardiomyopathy and peripheral polyneuropathy severity in patients with Glu89Gln mutation at the time of diagnosis

**DOI:** 10.1186/1750-1172-10-S1-P59

**Published:** 2015-11-02

**Authors:** Mariana Gospodinova, Stayko Sarafov, Velina Guergueltcheva, Andrey Kirov, Teodora Chamova, Albena Todorova, Ivailo Tournev, Stefan Denchev

**Affiliations:** 1 Medical Institute of Ministry of Interior, Clinic of Cardiology, 1606, Sofia, Bulgaria; 2 University Hospital Alexandrovska, Clinic of Neurology, 1431, Sofia, Bulgaria; 3 University Hospital Sofiamed, Neurology Department, 1528, Sofia, Bulgaria; 4 Genetic lab, Genika, 1113, Sofia, Bulgaria

## Background

Hereditary transthyretin – related amyloidosis (ATTR), associated with Glu89Gln mutation is characterized by mixed phenotype – cardiac and neurological and an unfavorable prognosis.

## Patients and methods

We evaluated forty consecutive ATTR patients with Glu89Gln mutation, assessing cardiac and peripheral polyneuropathy involvement - 18 male, 22 female at a mean age of 57.6±6,7 years. A clinical examination, 12–channel ECG, conventional 2D, Doppler and tissue Doppler echocardiography were performed. A comprehensive clinical neurological assessment was performed, defining the stage of neurological disability according to Familial Amyloidotic Polyneuropathy scale. The routine neurological assessment consisted of evaluating the reflexes, sensation (touch pressure, pin-prick, vibration, joint position) and muscle weakness.

## Results

Median age of symptoms development was 52, 3±6, 4 years. In 17 (42,5%) patients the disease started with carpal tunnel syndrome. Sixteen patients (40%) had sensory-motor symptoms at presentation. The first symptoms of the disease were cardiac in 5 (12,5%). Two (5%) patients exhibited gastrointestinal symptoms first. Median (range) delay from symptom onset to diagnosis was 62 (5–149) months. Cardiomyopathy and peripheral polyneuropathy were evident at diagnosis in all patients. Symptoms from the autonomous nervous system were found in 26 (65%) patients. The kidney and liver tests were normal in all patients.

Echocardiography revealed an infiltrative cardiomyopathy with varying degrees of LV diastolic dysfunction – Grade 1 in 11 (27,5%) patients, Grade 2 in 12 (30%) and Grade 3 in 17 (42,5%) patients. A reduced LV ejection fraction was found in 9 (22,5%) of the patients, all with severe diastolic dysfunction. At the time of diagnosis 24 (60%) patients were in the 1st neurological stage, 5 (12,5%) in the 2nd stage and 11 (27,5%) in the 3rd stage.

## Conclusion

Despite the fact, that most of the patients presented with neurological symptoms, either from the peripheral polyneuropathy or carpal tunnel syndrome, we found more patients with severe heart involvement than with 3rd stage of the polyneuropathy at the time of diagnosis. Our findings imply that the patients with Glu89Gln mutation have a prolonged period of asymptomatic heart involvement and the symptoms are further concealed by the development of neuropathy, which impairs functional class assessment. An earlier identification of the cardiomyopathy is needed through close follow up of the patients and the mutation carriers.

